# Cigar-Specific Health Warnings: Attention, Recall, and Perceived Effectiveness Among Young Adult Users and Non-Users

**DOI:** 10.3390/ijerph21111442

**Published:** 2024-10-30

**Authors:** Elizabeth G. Klein, Anne E. Driscoll, Abigail B. Shoben, Joseph M. Macisco, Stephanie Pike Moore, Amanda J. Quisenberry, Erika S. Trapl

**Affiliations:** 1 College of Public Health, Ohio State University, Columbus, OH 43210, USA; driscoll.198@osu.edu (A.E.D.); shoben.1@osu.edu (A.B.S.); 2 Department of Population and Public Health Sciences, Boonshoft School of Medicine, Wright State University, Dayton, OH 45435, USA; joe.macisco@wright.edu; 3 Department of Population and Quantitative Health Sciences, School of Medicine, Case Western Reserve University, Cleveland, OH 44106, USA; snp39@case.edu (S.P.M.); erika.trapl@case.edu (E.S.T.); 4 Department of Health Behavior, Roswell Park Comprehensive Cancer Center, Buffalo, NY 14263, USA; amanda.quisenberry@roswellpark.org

**Keywords:** cigars, tobacco, health warning labels

## Abstract

Limited research has examined attention to these cigar-specific health warnings and their perceived effectiveness among young people. The objective of our study was to evaluate the attention to and perceptions of a set of cigar-specific health warnings among young adult tobacco users and non-users. Methods: Young adults ages 18–24 in Columbus, Ohio, were recruited into an eye-tracking experiment examining cigarillo packaging between May 2022 and February 2023. Participants (*n* = 124) were shown 12 unique, branded cigarillo packages featuring a rotation of four of the Food and Drug Administration’s mandated health warnings: (1) Cigar smoking can cause lung cancer and heart disease (“disease”); (2) tobacco smoke increases the risk of lung cancer and heart disease, even in nonsmokers (“nonsmokers”); (3) cigar smoking can cause cancers of the mouth and throat, even if you do not inhale (“inhale”); and (4) cigars are not a safe alternative to cigarettes (“alternative”). Software captured visual attention to each product package, including the health warning. Participants also ranked the most effective message to motivate people to quit; one week later, the participants (*n* = 118) self-reported unaided recall of the experiment. Results: Study participants were an average of 21.2 years old, 54.2% were female, 73.7% were White, 65.3% had some college education, and 26.3% reported tobacco use in the previous month. The health warning, “Cigar smoking can cause cancers of the mouth and throat, even if you do not inhale” was ranked the most effective cigar warning (41.5%) and drew the greatest proportion of visual attention (26.1%). More than half (52.5%) recalled details regarding the health warning messages one week following the experiment, with few recalling (17.7%) specific warning message themes. Conclusions: Understanding the best performing health warnings is a crucial strategy to share accurate information on the risks of tobacco use. Our findings suggest that the warning on cancer risk even without inhaling drew the greatest visual attention and highest rating of perceived effectiveness among this sample of young adult cigarillo users and non-users.

## 1. Introduction

Health warning labels on tobacco product packaging provide an opportunity for users and potential users to be exposed to health risk information [1]. The majority of tobacco warning label research has centered around cigarettes [2], with less evaluation of the Food and Drug Administration’s (FDA’s) current mandated health warnings for other tobacco products. The category of cigars includes a diversity of products that vary in size, flavor, types of packaging, and tip and style [3]. Due to a legal decision in 2023, the FDA’s authority applies to all cigar products except premium cigars [4]. Cigarillos can be characterized as a mass merchandise midsize tobacco product that dominates more than 94% of the United States market [5]. Cigarillos have a 1.7% prevalence of use among adults [6]. There is disproportionate use by young people and marginalized communities, and the health risks of the use of these products are not well understood within these groups [7,8,9,10,11].

Health warnings are an important component of a broader tobacco risk communication strategy, as the FDA mandates that one of six health warning statements be present on the package label; the warnings statement themes include mouth/throat cancer, lung cancer/heart disease, the fact that they are not a safe alternative to cigarettes, secondhand smoke, risks of use during pregnancy, and addiction. Current evidence on health warnings more broadly recommends the use of graphic imagery, large visible size, and a regular rotation of messages [12,13]. For message content to effectively reach both users and non-users, it should be specific to the health risks and other consequences of using the product [7,14,15]. Yet few studies have evaluated the perceptions of the currently approved cigar warnings [16,17,18]. Young adults and current cigarillo users are known to misperceive cigarillos as less harmful relative to cigarettes [19,20], and specific diseases resulting from cigar use have been underestimated in some samples, suggesting that knowledge of the harm may not be well understood [19]. These misperceptions underscore the importance of effective health warning messages to correct this miscomprehension.

Given less robust examination of the health warning messages specified for cigars and their perceptions, the purpose of the present study was to evaluate the attention to and perceptions of a set of cigar-specific health warnings among young adult tobacco users and non-users. Our research question was to evaluate whether there are differences in the visual attention and recall of cigar-specific health warnings.

## 2. Materials and Methods

### 2.1. Study Sample

A convenience sample of young adults in Columbus, Ohio, was recruited between 9 May 2022 and 1 February 2023 through social media and online outlets popular with the target age group (e.g., Reddit, Facebook, Instagram, ResearchMatch.org, GroupMe, etc.). A detailed description of study recruitment is published elsewhere [21]. Study eligibility criteria included (1) being between the ages of 16 and 28 years of age; (2) self-report of having no chronic eye diseases known to interfere with calibration on eye-tracking equipment, such as glaucoma, regression lenses, or other similar issues; (3) being willing to participate in person in a session held in Columbus, Ohio; and (4) willing to complete online informed consent and participate in person in the study protocol. A total of 1313 unique individuals were screened, 356 of whom met eligibility criteria and 124 of whom were enrolled and computer-randomized to one of three unblinded experimental conditions using eye tracking to examine package images for flavored, unflavored, or a mixture of both flavored and unflavored cigarillos. All participants were invited to complete an online follow-up survey one week after completing the in-lab procedures.

All participants in this study provided written informed consent as part of the web-based screening process once eligibility had been determined. The study protocol was reviewed and approved by The Ohio State University (OSU) Institutional Review Board (IRB) and was registered at Clinictrials.org (ID: NCT04358705).

### 2.2. Experimental Procedures

Participants attended a single in-person eye-tracking session in a private office space located in Columbus, Ohio, at the Center for Tobacco Research (https://cancer.osu.edu/for-cancer-researchers/research/research-institutes-and-centers/center-for-tobacco-research, accessed on 4 May 2024). Participants were seated in a chair at a typical viewing distance from a monitor equipped with an infrared camera to continuously monitor gaze and capture precise eye movements using the Smart Eye Aurora (Smart Eye AB, Gothenburg, Sweden) [22]. A standard calibration procedure was completed to ensure data quality.

**Figure 1:**
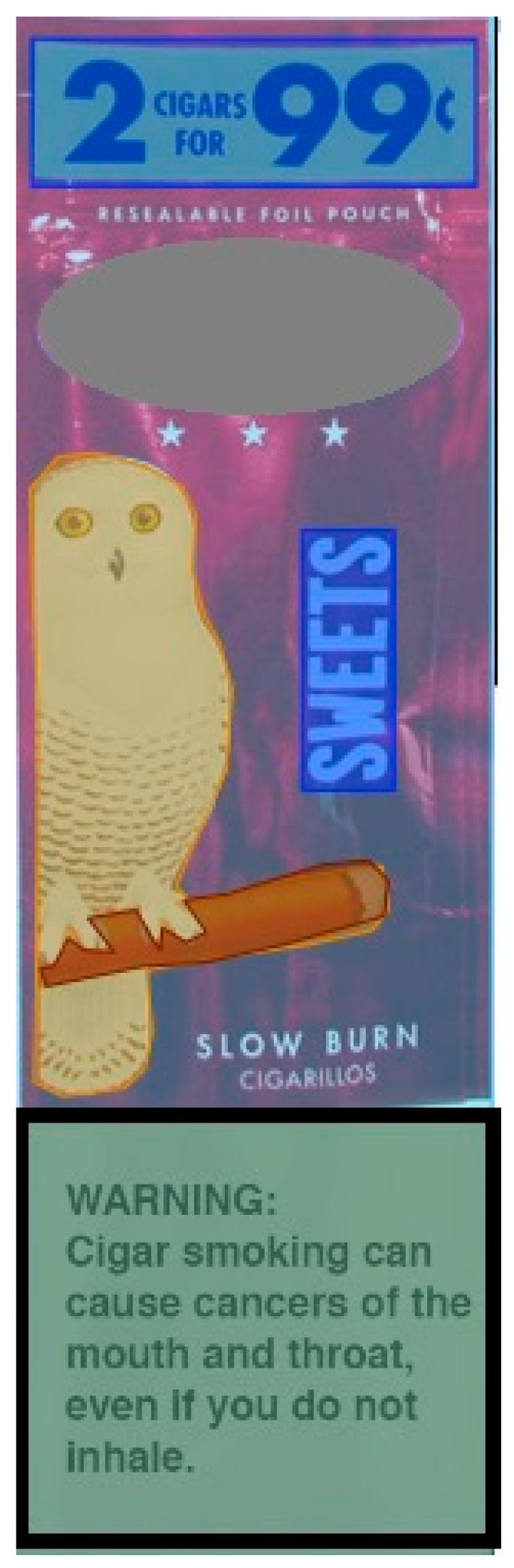
Areas of interest (AOIs) on a cigarillo package; health warning outlined in black.

In a randomized order, participants were presented with pictures of 12 currently available U.S. brands of cigarillo packages. Concurrently with the cigarillo package, one of four health warning labels was also presented (an example of a cigarillo package–warning label pair is shown in Figure 1). Each image pair was displayed on screen for 6 s.

This fixed interval was based on previous eye-tracking studies that reported a mean viewing time of 5–10 s [23,24]. Following visualization of a package, an on-screen image re-centered the participant’s gaze for standardization prior to viewing the next image. Participants were shown images in a random sequence.

For this experiment, images of foil 2-packs of real-world cigarillos were identified from 12 current cigarillo brands. A graphic designer modified the product packages for visual consistency so that all products were shown with identical pricing information (e.g., 2 for 99 cents), and one of four selected text-only health warning messages was fixed on the bottom portion of the package for all products. The health warning messages were in accordance with the FDA’s required warning statements [25]: (1) Cigar smoking can cause lung cancer and heart disease (“disease”); (2) tobacco smoke increases the risk of lung cancer and heart disease, even in nonsmokers (“nonsmokers”); (3) cigar smoking can cause cancers of the mouth and throat, even if you do not inhale (“inhale”); and (4) cigars are not a safe alternative to cigarettes (“alternative”). All study conditions viewed all four health warnings. The remaining two FDA-approved health warnings were excluded from the present study due to tailoring for health impacts of pregnancy or not being cigar-specific (“Cigar use while pregnant can harm you and your baby,” and “This product contains nicotine. Nicotine is an addictive chemical.”). Following the experimental procedures, participants self-reported their response to the survey items regarding the experimental content.

### 2.3. Metrics and Statistical Analysis

For the eye-tracking metrics, dwell times, sometimes referred to as total fixation or fixation length, were quantified by eye-tracking equipment for specified areas of interest (AOIs). The dwell time metrics were captured by the equipment and then aggregated for the predefined AOI. For the present analysis, the AOI was the health warning, shown in Figure 1 outlined in black. For the primary outcome, the proportion of viewing time on the health warning was calculated as average dwell time across the themes of the health warning AOI divided by the dwell time on the entire cigarillo package. Across the cigarillo packages, flavor names represented 4.3% of the total package size when present; brand names were larger, at 9.8% of the space; price promotions took up 9.3% of the space; health warnings predominantly took up 28.8%; and cigarillo imagery comprised 7.8%, when present.

In a self-administered survey following the experiment, participants reported demographics, including use of cigarillos or other tobacco in the previous month, susceptibility to using cigarillos in the coming month [26], and other demographics. Participants were given an unaided, open-ended response to describe content they viewed during the experiment: “Please list anything you remember about the content you viewed during the experiment; provide as much detail as you can.” Participants were also asked to recall health warnings shown in the experiment with the question, “Which of the following statements was on the cigar package you just saw (select all that apply)?”, with the response categories including all four health warning messages displayed in the experiment. Participants were asked to rank order the four cigar warnings: “Rank these messages from most effective (1) to least effective (4) for motivating people to quit or preventing people from smoking cigarillos.” In a follow-up online survey distributed via email one week after the in-lab session, participants were prompted, unaided, to recall any of the experimental content with the following statement: “Please list anything you remember about the products, text, or images you viewed on screen; provide as much detail as you can.”.

Data for all study conditions were collapsed across randomized groups, as these were post hoc analyses and all conditions received all health warning exposures. Descriptive statistics were qualitatively compared across conditions. Eye-tracking results were then examined for differences based on cigarillo use in the previous month (yes/no). For the self-reported health warning ranking task, the highest-ranking message for perceived effectiveness was summarized overall and examined for differences in those who had used tobacco in the previous month compared to non-users. All quantitative analyses were conducted using chi-square and *t*-tests in SAS 9.4. (SAS Institute; Cary, NC, USA) The qualitative statements were reviewed by two trained coders (E.G.K., A.E.D.) to be categorized dichotomously (present/absent) for any reference to the health warning message content or any other information related to the health warning. The Cohen’s kappa for interrater reliability was 0.88, indicating excellent agreement.

## 3. Results

Of the study participants (*n* = 118) who completed all eye-tracking procedures and the post-experimental survey, they were 21.2 years of age on average (range: 17–28 years), 54.2% identified as female, 77.1% identified as heterosexual, 13.6% identified as bisexual or pansexual, 73.7% were White or Caucasian, 7.6% were Black or African American, 3.4% were Hispanic, 65.3% had some college education or an associate degree, and 26.3% had used tobacco in the previous 30 days. The distribution of age, gender, racial or ethnic identity, and use of cigarillos in the previous 30 days was similar across the experimental study conditions.

**Figure 2:**
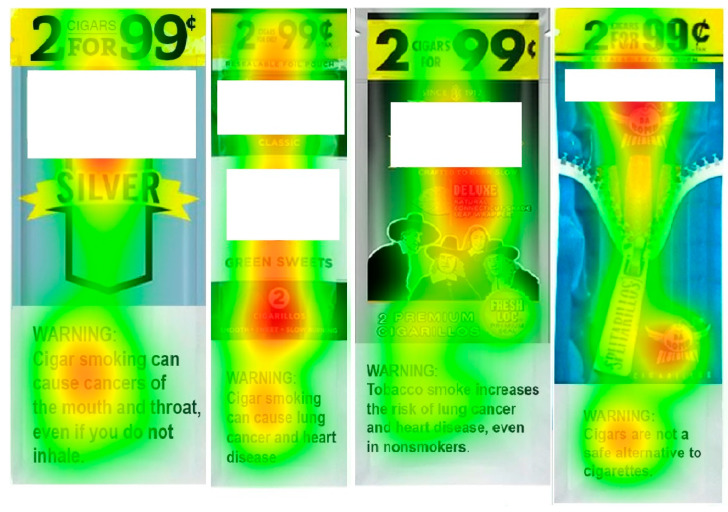
Heat maps of visual attention to varying health warning label messages. Green-yellow-red indicates least to greatest dwell time.

The mean proportion of time spent viewing the health warnings (shown in Table 1) was greatest for “inhale” (26.1%), followed by “disease” (20.4%), “nonsmokers” (17.6%), and “alternative” (16.2%). A heatmap of visual attention to the varying health warning messages, aggregated across all users, is shown in Figure 2. For the “inhale”, “disease”, and “alternative” messages, non-users had a greater proportion of visual attention compared to users. Participants endorsed “inhale” as the most effective cigar warning (41.5%) with no significant difference by user group.

More than half (52.5%) of the participants recalled the cigar warning messages one week following the experiment with general statements like, “Each product had a warning at the bottom”, or “FDA warning label”. A minority of the participants (17.7%) recalled specific warning messages with statements like, “Cigars are not a good substitute for cigarettes” or a reference to a message about “cancer”.

## 4. Discussion

The FDA-mandated cigar warning, “Cigar smoking can cause cancers of the mouth and throat, even if you do not inhale” received the greatest visual attention and the highest rating of perceived effectiveness among this sample of young adult cigarillo users and non-users. Understanding visual attention to health warnings is an important first step in informing users and non-users about the product [27], and visual attention is the first part of the behavioral cascade toward behavior change [28,29].

As noted by Cornacchione and colleagues, limited studies have focused on ways to optimize warnings for cigars, and warning statements that are demonstrated to improve awareness or knowledge about the harms of smoking cigars is an important outcome for the FDA in assessing warning effectiveness [14]. The current cigar health warnings do not meet the recommended best practices for health warnings due to a lack of imagery, the relatively small proportion of the package size, and the limited rotation of messages [1,12]. As noted by Popova and colleagues, the evolving landscape of other tobacco products requires continuous research on how to best communicate their harms, both absolute and relative to cigarettes [30].

Health warnings are a key intervention strategy for informing the public on the health risks associated with tobacco use. Enhancing the current health warnings is especially important for cigarillos given the popularity with youth and young adults and the known underestimation of health risks with use due to less frequent product use [20,31]. As noted by Cornacchione and colleagues, long-term health effects may be a lesser concern to young adults, particularly if they view their infrequent use as less risky [7]. Data from the PATH study found that noticing cigarillo warnings is associated with stronger perceived harm, so enhancing attention to health warnings has the potential for public health benefits [31]. Examination of the warning messages to increase their salience is a critical component of informing public health policy to prevent or reduce tobacco use [31,32].

## 5. Conclusions

Examination of the attention to and perceptions of cigar-specific health warnings among young adults adds important context to the current FDA-mandated labels on cigarillo packaging. Eye-tracking metrics help to confirm the visual attention to specific warning messages, as well as the rate of effectiveness. Continued efforts to use research to optimize health warnings on other tobacco products can yield important information for policy decisions that can have important impacts on public health.

## Figures and Tables

**Table 1:** Visual attention to cigar health warnings by message, perceived effectiveness, and user status (*n* = 118).

Cigar-Specific Warning Statement ^a^	% Top Rank (*n* = 118) ^b^	Users (*n* = 31)	Non-Users (*n* = 87)	% Dwell Time (*n* = 118)	Users (*n* = 31)	Non-Users (*n* = 87)	*p*-Value
Cigar smoking can cause cancers of the mouth and throat, even if you do not inhale (“inhale”).	49 (41.5%)	16 (51.6%)	33 (38.0%)	26.1%	18.9% (14.4)	29.1% (16.5)	0.002
Cigar smoking can cause lung cancer and heart disease (“disease”).	18 (15.3%)	2 (6.5%)	16 (18.4%)	20.4%	13.4% (13.0)	16.2% (16.2)	0.001
Tobacco smoke increases the risk of lung cancer and heart disease, even in nonsmokers (“nonsmokers”).	40 (33.9%)	10 (32.3%)	30 (34.5%)	17.6%	15.5% (17.0)	18.4% (15.4)	0.358
Cigars are not a safe alternative to cigarettes (“alternative”).	11 (9.3%)	3 (9.7%)	8 (9.2%)	16.2%	12.1% (11.0)	17.9% (15.8)	0.049

^a^ Proportion of average visual attention across each health warning theme. ^b^ Participants ranked from highest to lowest (1–4) for most effective health warning.

## Data Availability

De-identified study data are available from GitHub at https://github.com/klein232/CFLASH.git (accessed on 28 June 2024).
